# Translation elongation: measurements and applications

**DOI:** 10.1080/15476286.2025.2504727

**Published:** 2025-05-16

**Authors:** Leslie Watkins, Mulin Li, Bin Wu

**Affiliations:** a Department of Biophysics and Biophysical Chemistry, Johns Hopkins University Johns Hopkins University School of Medicine, Baltimore, MD, USA; b Center for Cell Dynamics, Johns Hopkins University School of Medicine, Baltimore, MD, USA; c Solomon Snyder Department of Neuroscience, Johns Hopkins University School of Medicine, Baltimore, MD, USA

**Keywords:** Translation elongation, ribosome, single molecule, ribosome profiling, codon optimality, RNA modification

## Abstract

Translation converts genetic information in mRNAs into functional proteins. This process occurs in four major steps: initiation, elongation, termination and ribosome recycling; each of which profoundly impacts mRNA stability and protein yield. Over recent decades, regulatory mechanisms governing these aspects of translation have been identified. In this review, we focus on the elongation phase, reviewing the experimental methods used to measure elongation rates and discussing how the measurements shed light on the factors that regulate elongation and ultimately gene expression.

## Introduction

Translation, the final step of the central dogma, converts mRNA-encoded genetic information into functional proteins using ribosomes. This process consists of four stages: initiation, elongation, termination and ribosome recycling, each contributing to the overall protein output. Initiation, which determines whether and how frequently ribosomes are loaded onto transcripts, is often considered the key regulatory step of translation and has been extensively reviewed elsewhere [1–7]. However, growing evidence suggests that translation elongation plays a crucial role in ensuring the proper synthesis of functional proteins. This review focuses on mammalian translation elongation, highlighting experimental methods for measuring elongation speed and their applications in uncovering regulatory mechanisms.

**Figure 1.**
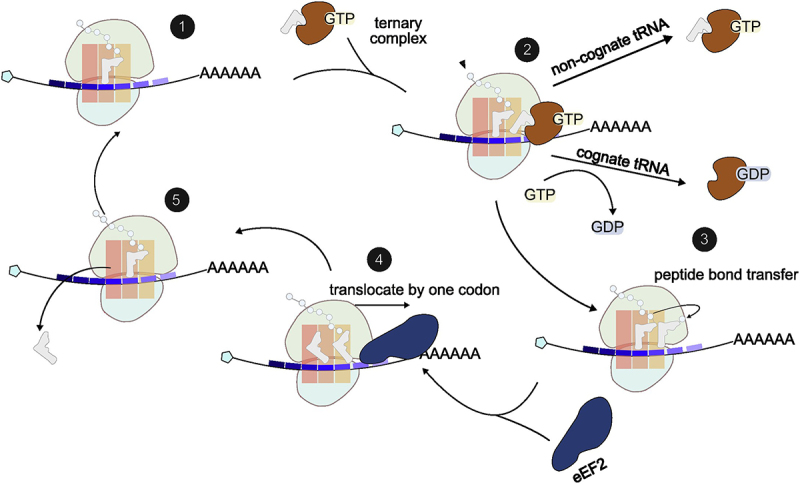
Overview of translation elongation cycles. Structural basis of eukaryotic elongation is extensively reviewed elsewhere [15–18]. Briefly, an elongation cycle starts with a charged aminoacyl-tRNA positioned at the P site of 80S ribosomes. A G-protein eEF1A delivers an aminoacyl-tRNA to the A-site of ribosomes. Upon cognate codon-anti-codon pairing, GTP-hydrolysis triggers the release of eEF1A from ribosomes, locking tRNA into the A-site. Next, the peptidyl transferase centre (PTC) catalyzes the peptide bond formation, transferring the nascent peptide from the P-site tRNA to the A-site tRNA. This process induces ribosome rotation, forming a hybrid state of E/P and P/A state tRNA. eEF2, a GTPase, binds to the rotated ribosome and translocates the ribosome forward by one codon, shifting tRNAs from site A to P and site P to E. eEF2 restores the ribosome to its original conformation, making the A-site available for the next tRNA.

During elongation, ribosomes add amino acids to the growing nascent peptide in a conserved cyclic process guided by the codon template on an mRNA. Each elongation cycle consists of three sub-steps: decoding, peptide bond formation and translocation, all catalysed by ribosomes with assistance from essential elongation factors. In the decoding step, a cognate aminoacyl-tRNA is selected and positioned in the ribosomal A-site, mediated by the ribosomal decoding centre and elongation factor eEF1A [8–10]. This is followed by peptide bond formation between the nascent peptide and the incoming aminoacyl-tRNA, catalysed by the ribosome’s peptidyl transferase centre [11–13]. Finally, elongation factor eEF2 facilitates ribosome translocation, moving the ribosome one codon towards the 3’ end of mRNA [14]. The cycle repeats until a stop codon is reached, triggering ribosome termination and recycling (Figure 1).

Beyond its primary role in protein synthesis, translation elongation serves as a key sensor of cellular states and a quality control checkpoint for both proteins and RNA. In a translation cycle, ribosomes spend most of their dwell time in the elongation phase, during which they unwind mRNA secondary structure, remove bound proteins, and detect transcript abnormalities to ensure accurate expression of mRNA. Given its frequent interaction with cellular factors, elongation serves as a rheometer that reflects the physiological state of the cell. Increasingly, ribosomes are recognized as signalling hubs that monitor transcriptome integrity and regulate cellular homoeostasis [19]. Elongation kinetics is a key metric that influences cellular decisions. For instance, slow ribosomes may indicate overall nutritional status. Depletion of specific amino acids may lead to the ribosome stalling at corresponding codons and result in ribosome collisions. Stalled ribosomes are sensed by ribosome quality control (RQC) factors, and prolonged stalling can trigger ribosome removal [20]. In some cases, transient and programmed elongation pauses are necessary for proper protein localization, such as ensuring correct integration of membrane proteins into lipid bilayers and proper targeting of organelle proteins [21,22]. Furthermore, codon-dependent variations in elongation speed have been implicated in protein folding in bacteria, although whether similar mechanisms exist in higher eukaryotes remains unclear [23].

The speed of elongation is tightly regulated and influenced by multiple factors. On a global scale, aminoacyl-tRNA availability and post-translational modifications of ribosomes and translation elongation factors directly impact elongation rates across all transcripts [24]. External conditions, such as nutrients and growth factors, generally enhance translation, whereas cellular stress tends to inhibit it. At the individual mRNA level, elongation speed is modulated by factors including codon usage, mRNA secondary and tertiary structures, nucleotide modifications and interaction with nascent peptides. Understanding these regulatory mechanisms provides critical insights into gene expression and protein synthesis.

A fundamental question in translation research is identifying factors that modulate total protein output. However, dissecting the contribution of translation elongation remains challenging, as the final protein output is impacted by multiple processes, including transcription, translation and mRNA stability. To advance our understanding of elongation regulation and its impact on cellular processes, methods to directly measure translation elongation speed are essential. Here, we will review techniques used to probe elongation kinetics and discuss how they have been applied to uncover intriguing elongation-coupled cellular processes.

## Methods to measure translation elongation

Accurately measuring elongation speed is crucial for understanding its regulation. Over the past few decades, diverse *in vitro* or *in vivo* methods have emerged, uncovering previously uncharacterized regulatory mechanisms. These techniques generally probe elongation speed by measuring ribosome occupancy, mature protein abundance, or nascent peptides emerging from elongating ribosomes. In this section, we explore various approaches for quantifying translation elongation rates (Table 1), highlighting their strengths, limitations and contributions to our understanding of elongation regulation.

**Table 1.** Elongation speed measured by various *in vitro* and *in vivo* methods.

Method	Measured elongation speed (aa/s)
smFRET	0.4
Translation competent lysate	0.7–1.5
Ribosome Profiling	3–5
SINAPs	4.15–4.8
SINAPs with socRNA	2.5

### In vitro single-molecule imaging of translation elongation

**Figure 2.**
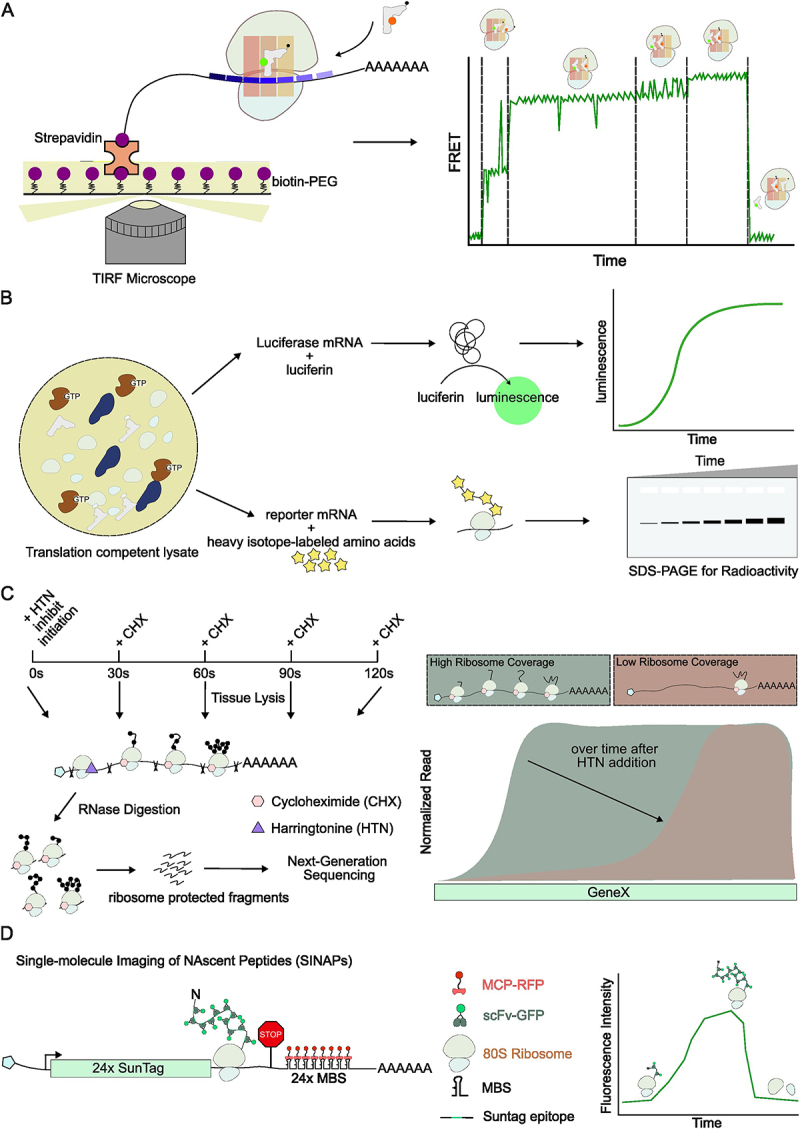
Methods for measuring elongation rates. (A). single-molecule FRET to measure a single elongation cycle. Aminoacyl tRNAs are fluorescently labelled and applied to ribosomes in translation-competent cell lysates. Distinct FRET states correspond to ribosome mechanics. This method measures elongation rate by measuring the time duration of FRET state cycles. (B). Translation competent cell lysate. mRNA templates are added to the lysate to initiate translation. Luciferase mRNAs can be used to probe elongation rate by measuring luminescence signal over time. To measure elongation rate of mRNAs lack of fluorescence or bioluminescence, mRNA templates are added to lysate with heavy isotope-labelled amino acids, and newly synthesized peptides can be visualized on SDS-PAGE. Accumulation of radioactive signal over time reports translation rate. (C). Ribosome profiling. This method determines gene-specific elongation rates by using harringtonine (HTN) to block initiation, followed by cycloheximide (CHX) to freeze elongating ribosomes at different time points. Sequencing ribosome-protected fragments maps ribosome positions on transcripts. The loss of ribosome coverage on transcripts over time reveals elongation rates. High sequencing depth is required for low-expression transcripts. The exact time course of CHX treatment may need optimization depending on gene targets. (D). Live-cell translation imaging. Single-molecule imaging of nascent peptides (SINAPs) utilizes an array of genetically encoded epitope tags and single chain variant fragments conjugated with green fluorescent proteins (scFv-GFP) to visualize nascent peptides, whereas single mRNAs are visualized via repeats of MS2 binding sites (MBS) in their 3’UTR and MS2 coat proteins fused to red fluorescent proteins (MCP-RFP). This method measures elongation rates by tracking either ribosome dwell time on a transcript or the dynamics of nascent peptide signal.

By fluorescently labelling translation machinery, such as tRNAs, ribosomal subunits or the initiation/elongation factors, *in vitro* single-molecule imaging enables direct observation of translation dynamics (Figure 2(A)). Single-molecule fluorescence resonance energy transfer (smFRET) is widely used to observe macromolecular conformation and has been used to study ribosome mechanics. Fluorescence labels can be strategically placed on either ribosomes or amino-acyl tRNAs (aa-tRNAs) without disrupting translation. Distinct FRET efficiencies and transitions reflect ribosomal conformational states during a elongation cycle [11,25–27]. Translation speed can be inferred by measuring the time duration of each FRET cycle, which was estimated to be ~0.4 amino acids per second (aa/s), notably lower than other reported elongation rates [25]. The discrepancy likely results from lower tRNA and elongation factor concentrations compared to cellular conditions. Despite this limitation, smFRET remains a powerful tool to investigate detailed ribosome mechanics.

### Translation elongation measurement in cell lysate

Translation-competent cell lysate derived from mammalian cells retains active translation machinery, enabling elongation measurement in a controlled setting. Cell lysates are typically treated with RNase to remove residual mRNAs so that fresh mRNA templates can be studied orthogonally (Figure 2(B)). Using mRNAs encoding for luciferase can estimate elongation speed by dividing the open reading frame (ORF) length by the time of the first detectable luciferase signal [28]. With this method, reported elongation speeds are ~0.7 aa/s in Hela extract and ~1.5 aa/s in rabbit reticulocyte lysates (RRL), significantly slower than most live-cell measurements [29]. This method includes contributions from initiation, elongation and protein folding, potentially underestimating elongation speed. Additionally, dilution of translation machinery in lysates may also contribute to the slower measured rate.

Heavy isotope-labelled amino acids (i.e. ^35^S-methionine) were used to label nascent peptides of any generic mRNA templates whose products do not produce fluorescence or luminescence. It offers moderate temporal resolution but suffers from low signal-to-noise ratio and semiquantitative output [28]. When combined with mass-spectrometry, heavy isotope labelling via stable isotope labelling by amino acids in cell culture (SILAC) enables global translation elongation rate measurement [30].

### Ribosome profiling

Ribosome profiling maps ribosome footprints on mRNAs, providing codon-level resolution of ribosome positions [31–33]. To measure elongation speed, cells are treated with harringtonine, which inhibits translation initiation while allowing elongating ribosomes to continue translating until they ‘run-off’ from the transcript (Figure 2(C)). By profiling ribosome occupancy at different time points after harringtonine treatment, ribosome elongation speed can be measured. This approach covers the entire transcriptome, capturing both global and gene-specific elongation rates. The reported average elongation speed ranges from 3 to 5 aa/s. However, accuracy may be influenced by drug diffusion rate and potential biases towards abundant transcripts.

### Translation imaging of single mRNAs in live cells

Recently, a series of methods were developed to image translation in live cells at single-mRNA resolution [34–38]. These techniques, collectively referred to as the single-molecule imaging of nascent peptides (SINAPs), utilize reporters containing tandem epitope tags that bind single chain variant fragments conjugated with a green fluorescent protein (scFv-GFP) upon emerging from ribosomes, illuminating active translation sites (Figure 2(D)). At the same time, these mRNAs contain MS2 binding sites (MBS) in the 3’ untranslated region (UTR) that are labelled by MS2 coat proteins fused to red fluorescent proteins (MCP-RFP) (Figure 2(D)). By tethering mRNA to the plasma membrane, mRNAs and their translation can be tracked for hours with total internal reflection fluorescence microscopy (TIRFM) [37–39].

SINAPs can measure the translation elongation rate when a single ribosome translates an mRNA at a time. The elongation speed is estimated by dividing the ORF length by the time-lapse of the nascent peptide signal, yielding a rate of 4.2 aa/s [40]. However, weak fluorescence signals from single ribosomes make it challenging to accurately define translation start and stop times. Moreover, translation on most mRNAs occurs in polysomes, where multiple ribosomes translate on a single mRNA simultaneously, complicating accurate calling of translation initiation and termination. The solution is harringtonine ribosome run-off [41]. Elongation rates can be determined from the survival time of translation sites after harringtonine treatment [38,40,42,43]. To average out the variance in measurement caused by drug diffusion, the elongation speed of ORFs with different lengths can be measured by fitting the ORF length as a linear function of run-off time [39]. By tethering mRNAs to different subcellular compartments, SINAPs can reveal elongation rates of locally translated transcripts [41].

To accurately measure the elongation speed of ribosomes, the Tanenbaum group developed a stopless-ORF circular RNA system (socRNA) to trap ribosomes in a circularized mRNA, where they translate indefinitely [44,45]. The socRNA encodes SunTag to visualize translation and an AlfaTag to tether nascent peptides to the plasma membrane for long-term tracking [46]. This enables monitoring of a single ribosome translating hundreds of thousands of codons. By analyzing translation site intensity over time, the translation elongation speed can be accurately measured. Measuring single ribosomes revealed significant heterogeneity in elongation rates. Notably, in cases where multiple ribosomes are trapped in a socRNA and collide due to the presence of a stalling sequence, they cooperate to increase the elongation speed, a previously unrecognized phenomenon [45]. SINAPs enables elongation measurements in living cells; however, it relies on reporter transcripts. Endogenous messages may display higher variation or dynamic regulation that is not captured by SINAPs reporters.

## Methods in action: investigating elongation kinetics

**Figure 3.**
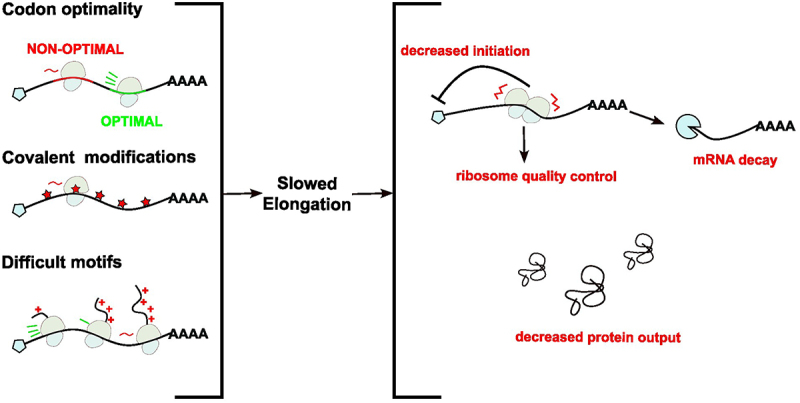
Factors affecting elongation rates. Non-optimal codons (red) may be translated slower than optimal codons (green). m1Ψ modified bases (red) may cause elongation speed to slow compared to unmodified ones. Besides mRNA sequence, peptides encoding polybasic residues cause elongation to slow and even stall. As a consequence of slow elongation, ribosomes can stall and collide. These collisions are cleared by the RQC pathway. Ultimately, slow elongation leads to decreased initiation, mRNA decay and decreased protein output.

As reviewed, numerous tools are available to measure elongation rates, varying in technical complexity, accuracy and spatiotemporal resolution. No single approach is universal for all biological questions. In this section, we will examine the practical application of these methods. We will discuss their strengths, limitations, and how they have been leveraged to uncover factors influencing elongation kinetics (Figure 3).

### Codon optimality

Codon optimality refers to differential decoding rates among synonymous codons, canonically contributed to variance in cognate tRNA abundance [47]. Optimal codons are decoded faster by abundant tRNAs, whereas non-optimal codons are slower due to the scarcity of their corresponding tRNAs. Because decoding accounts for over 50% of an elongation cycle [25], less abundant tRNAs take longer to be selected, slowing the elongation at those non-optimal codons. While ribosome profiling studies in yeast have shown that ribosomes dwell longer on non-optimal codons than optimal ones (reviewed extensively elsewhere [47–49]), evidence in mammals remains sparse and conflicting [50]. Most mammalian studies rely on ribosome profiling or single-molecule imaging, yet their findings do not agree on the contribution of codon optimality to elongation speed. Despite these inconsistencies, codon optimality appears to influence total protein output in mammalian systems [51,52]. This section discusses whether codon optimality affects elongation in mammals.

In a seminal study, Ingolia et al. employed harringtonine run-off combined with ribosome profiling to measure elongation rates across the transcriptome in mouse embryonic stem cells [33]. The authors used the tRNA adaptation index (tAI) to define codon optimality [53], which is based on tRNA abundance. Surprisingly, no significant differences in elongation rates were observed between codons with high and low tAI scores, both averaging 5.6 aa/s. This suggested that, unlike lower eukaryotes, tRNA abundance may not play a prominent role in mammalian translation elongation. However, this study used cycloheximide to arrest elongating ribosomes after harringtonine treatment, which may introduce biases. In yeast, cycloheximide does not arrest ribosome elongation uniformly across all codons, leading to inaccurate estimation of elongation rates. Future studies may use flash freezing to mitigate these biases [47,54,55].

To further investigate this, the Tanenbaum’s group applied single-molecule translation imaging with ribosome run-off to study the effects of codon optimality in U-2 OS cells [38]. This study did not use cycloheximide and compared elongation rates between a non-optimal Kif18b reporter and a codon-optimized BFP reporter, also based on tRNA availability. The authors found that the codon-optimized transcripts translated faster (4.9 aa/s vs. 3.3 aa/s), supporting the role of codon optimality. However, differences in encoded amino acid sequence between optimized and non-optimized reporters may confound these results. A more accurate approach would involve using synonymous sequences of the same encoded protein to isolate the effects of codon optimality.

A subsequent study by the same group employed circular single-molecule RNA reporters with synonymous ORFs [45], without using confounding translation inhibitors. This study found that non-optimal reporters exhibited a modest reduction in elongation rates (6.5% to 16.5%) on non-optimal reporters, suggesting that codon optimality has a minor but detectable impact on mammalian translation. While these findings contrast with stronger effects observed in yeast [50], they highlight the need for further investigation in mammalian systems.

Interestingly, while codon optimization may not significantly enhance elongation rates in human cell lines, it is well documented that codon optimization increases protein production in mammals [51,52]. This is likely due to an increase in mRNA stability and/or initiation rates [56–61]. The mechanisms coupling codon optimality to translation initiation and mRNA stability are still unknown, though *in vitro* experiments on RRL and mammalian cells suggest that the Ccr4-NOT complex may be involved [57,62].

In humans, the tRNA pool varies across cell types, proliferation and activation states [63–65]. This poses an interesting question: do different cell types adapt their tRNA pools to optimize translation of their specific transcriptome? Most studies on codon optimality have relied on precalculated tRNA abundances rather than directly measuring tRNA levels in a specific cell type. Therefore, reporters designed to study codon optimality may not reflect actual tRNA availability in the tested cells, potentially explaining the modest impact observed on elongation rates. Future studies should first characterize codon usage in the relevant cell type to better assess the effect of codon optimality.

Conversely, an emerging body of work suggests that codon optimality in *E. coli* and yeast may not depend on tRNA abundance but rather on specific tRNA conformation within the ribosome [66]. GC content and mRNA secondary structure may also explain differences in protein output on synonymous codons [67,68]. These may all impact elongation rates in mammalian cells as well, regardless of tRNA abundance. Since the studies discussed here all designed reporters based on tRNA abundance or codon usage, this may explain why only a slight difference in elongation rates has been observed in mammalian cells.

### RNA covalent modification

RNAs are extensively covalently modified by diverse chemical groups. Depending on the location and properties of these modifications, they can alter canonical Watson–Crick base-pairing, non-canonical hydrogen bonding, and tertiary structures. In mRNAs, these modifications can affect translation decoding fidelity and speed. One well-studied modification is N1-methylpseudouridine (m^1^Ψ), originally identified in human rRNA [69]. Recently, m^1^Ψ has garnered attention for its usage in the COVID-19 mRNA vaccines due to its ability to reduce immunogenicity and enhance protein production [70]. Despite its widespread application, the effects of m^1^Ψ on translation fidelity and efficiency remain poorly understood. Because m^1^Ψ disrupts traditional Watson–Crick base-pairing, it can interfere with anticodon recognition, potentially influencing decoding fidelity and speed. This section discusses whether m^1^Ψ affects translation elongation rates in mammalian systems.

Using luciferase assays, numerous studies have shown that m^1^Ψ results in more functional proteins in cells [29,70–72]. The enhanced translational output is partially attributed to m^1^Ψ’s capability to evade innate immune activation and avoid inhibition of translation initiation [29]. Interestingly, m1Ψ increases RNA duplexes, potentially influencing elongation kinetics through stabilization of codon:anti-codon interactions [73,74]. However, whether m^1^Ψ directly affects elongation speed and fidelity remains debated. Svitkin *et al*. synthesized luciferase reporter mRNAs containing either m^1^Ψ or unmodified uridine and translated them using HeLa cell or RRL [29]. They report that in HeLa cell lysate, the elongation rates were 0.70 aa/s for unmodified mRNA and 0.56 aa/s for m^1^Ψ modified mRNA. In RRL, rates were 1.49 aa/s for unmodified and 0.96 aa/s for m^1^Ψ. These findings suggest that ribosomes translate m^1^Ψ modified mRNAs more slowly. However, several caveats must be considered. First, *in vivo* measurements report much faster elongation rates (~3–5 aa/s) [33,39], while these values are five-fold lower when measured with lysates. Second, luciferase assays only measure functional protein, and cannot separate other factors such as initiation rates, mRNA stability and protein misfolding.

To further investigate m^1^Ψ’s effect on elongation, Svitkin *et al*. utilized [^35^S]-methionine pulse-labelling assay in RRL [71]. The authors used polysome fractionation to directly extract nascent peptides associated with mRNAs and quantify [^35^S]-methionine incorporation in the whole polysome fraction. They found m^1^Ψ-modified mRNAs enhanced [^35^S]-methionine incorporation compared with unmodified ones. Assuming initiation rates and mRNA stability are unaffected, this implies that elongation is slower on modified transcripts. A better measurement would be testing whether m^1^Ψ mRNA is enriched in heavier polysome fractions. However, the authors did not report this information. Additionally, they observed more truncated peptides from m^1^Ψ modified mRNAs, possibly because of premature termination or elevated ribosomal collision. This aligns with recent evidence that m^1^Ψ can induce +1 ribosomal frameshifting in mammalian cells [72]. The studies reviewed here provide evidence that m^1^Ψ may slow down elongation, even though the current methods may not decouple effects from initiation, stalling, frameshifting or mRNA degradation. Future studies should employ a more direct elongation measurement that minimizes confounding factors, allowing for a more precise understanding of how m^1^Ψ affects ribosome movement.

### Translation through difficult motifs

Certain RNA structures and charged nascent peptides can pose significant challenges to ribosomes, often leading them to stall. For instance, a plethora of evidence shows poly-A sequences encoding a repulsive poly-lysine chain slow down and stall ribosomes, causing collision of trailing ribosomes [43,75–77]. In humans, these collisions are detected by the E3 ubiquitin-ligase ZNF598, triggering ribosome-associated quality control (RQC), reviewed extensively [78,79] to degrade nascent polypeptides and mRNA.

Difficult motifs are especially relevant in neurodegenerative diseases caused by repeat expansions. The GGGGCC hexanucleotide repeat expansion within the first intron of *C9ORF72* gene is the leading cause of familial amyotrophic lateral sclerosis (ALS) [80]. One potential pathogenic mechanism is the accumulation of toxic dipeptide repeats (DPRs) like poly-GA, GR and GP, produced from noncanonical translation of the expanded repeats [81,82]. However, it is unclear how different DPRs are synthesized. Here, we discuss studies evaluating the elongation kinetics along the three reading frames and how they contribute to dipeptide repeat synthesis.

Latallo et al. used ribosome run-off on GGGGCC repeat-containing SINAPs reporters to measure the elongation rate for different DPR [83]. They found that the GA frame exhibited normal elongation compared to typical proteins. In contrast, the GP and GR frames translated significantly slower. Since these reporters shared the same RNA sequence, the observed difference suggests that amino acid composition, rather than the RNA structure, affects elongation kinetics. The mechanism of slowed elongation in the GR frame is likely due to blockage of the ribosome exit tunnel [84], while GP can introduce high steric hindrance. In fact, when synthetic poly-GR was applied to cells, global elongation was slowed down [85], likely because poly-GR blocks the ribosomal exit tunnel *in trans* [84]. In addition, slow elongation may induce ribosome collisions, activating RQC factors, such as ZNF598 and Pelota, to modulate translation dynamics through repeats [83]. The methods used in these studies decouple initiation from elongation without the addition of cycloheximide. They further probe the mechanism of slow elongation by investigating ribosome collisions.

Interestingly, poly-proline and polybasic motifs can play a regulatory role within upstream open reading frames (uORFs). uORFs are short, alternative open reading frames upstream of the main start codon that can both increase and decrease the translation of downstream coding sequence (CDS) [86]. Ivanov *et al*. used polysome profiling to investigate elongation stalling in the uORF of antizyme inhibitor 1 (AZIN1) in mammalian cells [87]. AZIN1 regulates polyamine synthesis and is regulated by polyamine levels [87,88]. When polyamine is abundant, the uORF is translated, inhibiting initiation at the main ORF. Ivanov and colleagues showed through ribosome profiling that a PPW motif in the uORF causes ribosomal stalling. This stalling induces a ribosome queue in the uORF that surprisingly increases initiation at the uORF. Similarly, Bottorff and colleagues used reporter assays and kinetic modelling to come to a similar conclusion on UL4 mRNA: polyproline within a uORF induces ribosomal stalling and inhibits downstream initiation on the main CDS [89]. The same trend has also been observed with an IFI stalling sequence in a uORF of *CHOP* mRNA [90]. Thus, elongation stalling at difficult motifs within a uORF can inhibit initiation at the main open reading frame to regulate gene expression [88]. Together, these experiments provide strong evidence that polybasic and poly-proline sequences slow elongation rates in human cells.

### Tissue type and proliferation state

Most studies discussed thus far have been conducted on immortalized cell lines, which may not fully capture heterogeneous translation kinetics across morphologically and functionally distinct tissue types. To investigate how elongation kinetics vary between cell types, Gerashchenko *et al*. performed ribosome run-offs and profiling assays in mice [91]. The researchers administered harringtonine via tail vein injection, followed by cycloheximide treatment at different time points. After confirming that the treatment effectively inhibits translation, they observed tissue-specific differences in elongation rates. The liver exhibited the fastest elongation rate (6.8 aa/s), followed by the kidney (5.0 aa/s) and skeletal muscle (4.3 aa/s). Importantly, comparing the same genes across multiple tissue types revealed a consistent trend. Furthermore, elongation rates in the liver of 18-month-old mice were 20% slower than in 3-month-old mice, indicating that elongation slows with age. While harringtonine effectively decouples initiation from elongation, it remains unclear whether all tissues absorb the inhibitors at the same rates. Another caveat is that the analysed tissues consist of multiple cell types, each of which may have different elongation rates. Variations in tRNA abundance, ribosome heterogeneity and nutrient availability likely contribute to the difference in elongation rates observed across tissues. Despite these limitations, the study poses intriguing evidence that warrants further investigation into the underlying mechanisms.

## Discussion

Translation elongation is a highly regulated process that governs protein synthesis and serves as a surveillance mechanism to detect aberrant transcripts and nascent peptides. Elongation rates are intimately coupled with other regulatory pathways to modulate gene expression. Ribosome collisions on difficult motifs within uORFs can block initiation at downstream coding sequences [87–89]. In addition, collided ribosomes within main open reading frames can be sensed by GCN2, GIGY2 and 4EHP to inhibit translation initiation [92,93]. Translational stalling is a signal for mRNA decay via no-stop and no-go decay pathways [49,94]. However, one group found that a subset of mRNAs encoding C2H2 zinc finger proteins are stabilized in the presence of elongation inhibitors [95]. Changes in elongation rates therefore act as a signalling hub to regulate many stages of gene expression, playing a central role in total protein output (Figure 3) [23,92,95,96]. Accurate measurement of elongation rates is crucial for understanding translation in gene expression regulation. Various experimental approaches, including live-cell single-molecule imaging, ribosome profiling, *in vitro* translation and smFRET, offer unique insights into elongation dynamics. This review highlights the strengths, limitations, and pitfalls of these techniques. Specifically, harringtonine-based inhibition of initiation is an effective way to isolate elongation rates from other processes. An important insight from the review is that the elongation regulation pathways in bacteria or yeast do not always translate to mammalian systems, underscoring the need to investigate elongation rates in higher-order eukaryotes. As mRNA therapeutics continue to advance, understanding how RNA modifications and sequence elements influence translation elongation is critical for designing more effective mRNAs. The methodologies discussed here provide powerful tools for studying translation elongation, offering valuable insights that can drive the development of next-generation mRNA-based therapies.

## Author contribution statement

L.W. and M.L. drafted the initial document. L.W., M.L. and B.W. edited and approved the final manuscript.

## Data Availability

I confirm that I understand the terms of the share upon reasonable request data policy. I confirm that I have included a Data Availability Statement in my manuscript. Data Sharing does not apply to this article as no new data were created or analysed in this study.
